# Hmgb3 Is Regulated by MicroRNA-206 during Muscle Regeneration

**DOI:** 10.1371/journal.pone.0043464

**Published:** 2012-08-17

**Authors:** Simona Maciotta, Mirella Meregalli, Letizia Cassinelli, Daniele Parolini, Andrea Farini, Giulia Del Fraro, Francesco Gandolfi, Mattia Forcato, Sergio Ferrari, Davide Gabellini, Silvio Bicciato, Giulio Cossu, Yvan Torrente

**Affiliations:** 1 Stem Cell Laboratory, Department of Pathophysiology and Transplantation, Università degli Studi di Milano, Fondazione IRCCS Ca’ Granda Ospedale Maggiore Policlinico, Centro Dino Ferrari, Milano, Italy; 2 Dipartimento di Scienze Biomediche, Università di Modena e Reggio Emilia, Modena, Italy; 3 Dulbecco Telethon Institute and Division of Regenerative Medicine, San Raffaele Scientific Institute, Milano, Italy; 4 Department of Cell and Developmental Biology, University College London, London, United Kingdom; Telethon Institute of Genetics and Medicine, Italy

## Abstract

**Background:**

MicroRNAs (miRNAs) have been recently involved in most of human diseases as targets for potential strategies to rescue the pathological phenotype. Since the skeletal muscle is a spread-wide highly differentiated and organized tissue, rescue of severely compromised muscle still remains distant from nowadays. For this reason, we aimed to identify a subset of miRNAs major involved in muscle remodelling and regeneration by analysing the miRNA-profile of single fibres isolated from dystrophic muscle, which was here considered as a model of chronic damage.

**Methodology/Principal Findings:**

The miRNA-signature associated to regenerating (newly formed) and remodelling (resting) fibres was investigated in animal models of muscular dystrophies and acute damage, in order to distinguish which miRNAs are primary related to muscle regeneration. In this study we identify fourteen miRNAs associated to dystrophic fibres responsible for muscle regeneration and remodelling, and confirm over-expression of the previously identified regeneration-associated myomiR-206. In particular, a functional binding site for myomiR-206 was identified and validated in the 3′untranslated region (3′UTR) of an X-linked member of a family of sequence independent chromatin-binding proteins (Hmgb3) that is preferentially expressed in hematopoietic stem cells. During regeneration of single muscle fibres, Hmgb3 messenger RNA (mRNA) and protein expression was gradually reduced, concurrent with the up-regulation of miR-206.

**Conclusion/Significance:**

Our results elucidate a negative feedback circuit in which myomiR-206 represses Hmgb3 expression to modulate the regeneration of single muscle fibres after acute and chronic muscle damage. These findings suggest that myomiR-206 may be a potential therapeutic target in muscle diseases.

## Introduction

MiRNAs are a class of short non-coding RNAs that take part in mastering the balance of gene-regulating networks by binding to 3′UTR of target mRNAs and inhibiting their expression [1]. The disclosure of these small RNA molecules introduced a new labyrinthine dimension to gene regulation and gave the opportunity to deepen our understanding on many biological processes. MiRNAs were in fact demonstrated to be involved in most of biological events and to finely regulate them [1], [2]. Even more importantly, they were found dysregulated in many diseases [3], [4], [5], [6], [7], [8] and apart from their specific role, their normalization is considered a potential method of intervention in patients. Focusing on the involvement of miRNAs in muscle development and myogenesis, a restricted group of muscle-enriched miRNAs, also called myomiRs (miR-1, miR-133, miR-206 and miR-208), was demonstrated fundamental for muscle physiology and plasticity [9], [10], [11]. In line with a growing characterization of the myomiR network, researchers started to investigate the role of miRNAs in muscle degeneration, which turns out to be very challenging if considering the absence of an efficient therapy for patients affected by most of primary muscular disorders. A faithful model for studying muscle damage and regeneration, is represented by muscular dystrophies (MDs) since they are a group of diseases characterized by muscle wasting and weakness due to defects in structural proteins expressed in the skeletal muscle [12], [13]. In particular MDs are proposed as two-tiered diseases with acute large amount of myofibre necrosis resulting from growth spurts or damaging exercise, superimposed upon a background of a chronic low level of damage, with different factors contributing to these two situations [14]. When Eisenberg et al. analyzed the miRNome of muscle biopsies from patients affected by 10 major muscular disorders including MDs, 185 miRNAs were found to be differentially expressed in diseased muscle [15]. This evidence introduced an additional dimension to muscle degeneration, denouncing a strong participation of miRNAs in muscle degeneration. Soon after this discovery, Capogrossi and colleagues further investigated the role of miRNAs in muscle damage by studying their expression profile in an animal model of Duchenne Muscular Dystrophy (DMD), the mdx mouse, and of acute ischemia [16]. This study demonstrated a common signature of DMD and ischemic muscle, outlining three different families of DMD-signature miRNAs: inflammatory (miR-222 and miR-223), degenerative (miR-1, miR-29c, and miR-135a) and regenerative (miR-31, miR-34c, myomiR-206, miR-335, miR-449, and miR-494). Finally, Cacchiarelli and colleagues demonstrated the mis-regulation of several miRNAs in Duchenne condition (miR-1, myomiR-133, miR-29 and miR-30) as consequence of differential HCDAC nytrosilation state [17]. It is however important to underline that whole-muscle based-analyses, as studies mentioned above, supply a wide image of the miRNA-dysregulation associated to damaged muscle without performing a deep analysis of the muscular miRNAs involved in these processes. Dystrophic muscle is in fact characterized by inflammatory infiltrations and overcoming fibrotic- and adipose-tissue that progressively substitute skeletal muscle [13]. The present study was addressed to identify muscular miRNAs involved in muscle remodelling and regeneration whose in vivo modulation might help recovering from injury. In order to reach this aim, we initially profiled the miRNome of single muscle fibres isolated from the mdx mouse. The mdx mouse originates from a spontaneous mutation in exon 23 of the dystrophin gene resulting in a cyclic process of fibre necrosis and regeneration, and giving rise to progressive muscle wasting and replacement by connective and adipose tissue [18], [19], [20]. Although the mdx mutants have a belated onset of mild clinical symptoms compared with both Duchenne and Becker muscular dystrophy, they have several histopathological features in common [21], [22]. We thus validate our miRNome results in single muscle fibres isolated from other animal models of MDs such as the alpha-Sgca-null and the FRG1 over-expressing mice, respectively animal models of a limb-girdle 2D (LMGD-2D) [23], [24] and of Facioscapulohumeral dystrophy (FSHD) [25]. The α-Sgca null mouse represents the first engineered animal model for autosomal recessive MD with a primary sarcoglycan gene defect [23]. Like the mdx mouse, the main histological changes of α-Sgca-null muscles include extensive central nucleation, increased variability of muscle fibre diameter, and the persisted presence of degeneration and regeneration [23]. Regarding muscle impairment, absence of α-sarcoglycan was demonstrated to perturb more fast-twitch than slow-twitch fibres. The FRG1 over-expressing mouse is a transgenic mouse characterized by strong up-regulation of human FRG1, causing muscular dystrophy similar to FSHD (abnormal spinal curvature, progressive muscle dystrophy, skeletal muscle atrophy, differential involvement of muscle types similar to FSHD) [25]. Basing on several measurements (including fibre size variation, number of internal nuclei, percentage of connective tissue, number of necrotic fibres and number of regenerating fibres), this animal model demonstrated different distribution of muscle impairment. In particular, trapezius (TPZ) was defined as the most affected muscle in FRG1 high over-expressing mice and FSHD patients [25]. We also verified miRNAs associated to regenerating single muscle fibres after acute damage. Strategies that have been successfully used for this purpose include crush [26], freeze [27], or chemically induced injury [28]. Perhaps the most extensive and reproducible muscle injury is the delivery of cardiotoxin (CTX; purified from the venom of the Naja nigricollis snake) into the skeletal muscle of the mouse [28]. In this condition it is possible to induce local acute muscle damage with a muscle degeneration of about 80–90% [25] and a successive regeneration which restore muscle integrity and homeostasis [29]. We identify fourteen miRNAs associated to dystrophic fibres (miR-15b, miR-17, miR-21, miR-27a, miR-31, miR-128a, miR-142-5p, miR-199a-5p, miR-199b, miR199b*, miR-206, miR-221, miR-223 and miR-335-5p) that may mediate muscle regeneration and remodelling in animal models of MDs and acute muscle damage, and confirm over-expression of the previously identified regeneration-associated myomiR-206. Using a target-gene prediction-analysis with 6 different computational algorithms (PITA, TargetScan, PicTar, ElMMo, miRDb and miRanda), we identified and validated a functional binding site for miR-206 in the 3′ untranslated region (3′UTR) of an X-linked member of a family of sequence-independent chromatin-binding proteins (Hmgb3) [30]. Importantly, Hmgb3 expression was reduced during muscle regeneration concurrent with the up-regulation of myomiR-206 in dystrophic muscle. All these data demonstrate for the first time the involvement of Hmgb3, an Hmg-box family transcription factor, in muscle regeneration.

## Results

### The miRNA Expression Profile of Dystrophic Muscle Fibres Isolated from the Mdx Mouse

**Figure 1:**
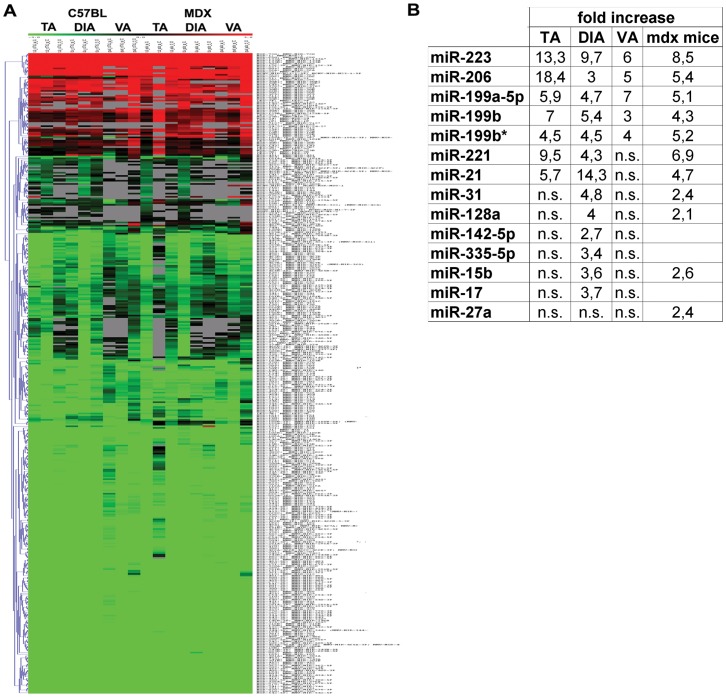
MiRNA profile of single fibres isolated from the mdx mouse. (A) Clustered heat map showing the expression ratios of miRNAs in TA, DIA and VA of age- and sex-matched c57bl (n = 3) and mdx (n = 3) mice. Expression data were normalized on Universal Reference. A total of 14 miRNAs were found over-expressed in dystrophic samples with distinction among myofibres isolated from different muscle type. (B) Fold change values of the 14 up-regulated miRNAs were reported in the table (ns = no significant).

Since the mdx mouse originates from a spontaneous mutation in exon 23 of the dystrophin gene and was previously demonstrated to determine muscle pathophysiology of DMD [18], [19], [20], we decided to investigated the role of miRNAs in muscle remodelling by evaluating the miRNome of single muscle fibres isolated from three different muscles (Tibialis Anterior, TA; Diaphragm, DIA and Vastus, VA) of this animal model. We selected different muscle tissues since the mdx mouse is characterized by a heterogeneous distribution of muscle wasting and weakness. As described, among the investigated muscles the DIA was the most affected showing a pattern of degeneration/regeneration, fibrosis comparable to the DMD muscle tissues [31]. Adult 3½ months-old mdx (n = 3) and c57bl (n = 3) mice were sacrified and single muscle fibres were dissected from TA, DIA and VA. The expression profile was performed by a miRNA expression microarray (Miltenyi Biotech, Bergisch Gladbach, Germany) containing probes matching for 584 mouse miRNAs from the miRBase database (Figure 1A). For the statistical analysis only miRNAs which had valid expression values in at least two samples per muscle type were considered and Two-class SAM (Significance Analysis of Microarrays) method was used.

Fourteen miRNAs were found dysregulated in dystrophic muscle fibres of the mdx mouse with differences linked to the originating muscle (miR-206, miR-199a-5p, miR-223, miR-199b, miR-199b*, miR-21, miR-221, miR-17, miR-15b, miR-31, miR-128a, miR-142-5p, miR-335-5p and miR-27a). Importantly, single muscle fibres isolated from the most affected DIA muscle showed 13 miRNAs out of the 14 dys-regulated miRNAs (Figure 1). Otherwise, single muscle fibres isolated from lower affected TA and VA of the mdx mouse were respectively associated to 7 (miR-206, miR-199a-5p, mir-223, mir-199b, miR-199b*, miR-21 and miR-221) and 5 (miR-206, miR-199a-5p, mir-223, mir-199b and miR-199b*) dysregulated miRNAs (Figure 1). The array analysis evidenced a dystrophic miRNA-signature not dependent to the muscle type of origin which include the regeneration-associated miR-206; miR-199a-5p, miR-199b, miR-199b* and miR-223. These data are in agreement with data already published on the involvement of miRNAs in mdx and DMD muscle [15], [16], [17]. Otherwise, the expression profile of miR-21 and miR-221 was strictly related to the muscle type of origin (TA and DIA) (Figure 1). MiR-15b, miR-17, miR-31, miR-128a, miR-142-5p and miR-335-3p were specifically induced in single muscle fibres of dystrophic DIA (Figure 1). Finally, miR-27a was only over-expressed when the statistical analysis was performed independently of the muscle type (Figure 1).

### Validation of Microarray Results by Absolute Q-PCR Analysis

Data obtained by the microarray analysis were validated by absolute Q-PCR analyses. Absolute quantification confirmed common up-regulation of miR-206, miR-199a-5p, miR-223 and miR-199b* in all mdx single fibres tested (Figure S1). MiR-199b dysregulation was statistically significant only in single muscle fibres of dystrophic TA (Figure S1A), nevertheless an increment was observed in fibres of dystrophic DIA and VA. A more accurate and sensible quantification of miRNAs by Q-PCR analysis, extended the miRNA signature common to dystrophic single fibres of TA, DIA and VA to miR-128a, miR-31 and miR-142-5p (Figure S1). Importantly, up-regulation of miR-128a and miR-31 in dystrophic single muscle fibres is strongly supported by their recently discovered involvement in MDs and myogenesis [32], [33]. As shown in Figure S1, dysregulation of miR-15b was extended to TA of the mdx mouse and higher expression levels of miR-221 and miR-335-5p were confirmed in dystrophic DIA. Finally, according to the array analysis, miR-27a over-expression was statistically significant only when the analysis was performed independently of the muscle type. In contradiction with the array analysis, Q-PCR experiments evidenced i) up-regulation of miR-21 in single muscle fibres isolated from VA and DIA of the mdx mouse; ii) up-regulation of miR-221 limited to dystrophic DIA; and iii) down-regulation of miR-17 in all dystrophic single muscle fibres analyzed (more than 30 folds) (Figure S1). In conclusion, a more sensible and specific quantification of miRNAs by absolute Q-PCR analysis highlighted common up-regulation of miR-206, miR-223, miR-199a-5p, miR-199b*, miR-27a, miR-128a, miR-31 and miR-142-5p, and down-regulation of miR-17 in dystrophic fibres isolated from TA, DIA and VA of the adult mdx mouse (Figure S1). Otherwise the expression profile of miR-15b, miR-221, miR-21, miR-199a and miR-335-5p was strictly related to the muscle-type considered (Figure S1).

Absolute quantification was also performed for miR-1, miR-133 and miR-181 because their expression profiles were formerly investigated in dystrophic muscle and their dysregulation in the mdx mouse was often controversial [10], [16], [17], [34]. In particular, their expression profiles seemed to strongly depend on the muscle type and age of the mdx mouse [10], [16], [17], [34]. When we quantified these miRNAs in muscle fibres isolated from TA, DIA and VA of adult mdx mice (3½ m-o; n = 10), we did not evidence differences between dystrophic and control muscle fibres (Figure S1), as previously highlighted by the array analysis.

Since quantification of miRNAs by absolute Real-time PCR (Q-PCR) analysis represents an innovative and original method, we chose a relative quantification approach (qRT-PCR) to confirm data obtained. With this approach the expression of miRNAs was compared to invariable RNAU6 (normalizing factor) using a comparative C_T_ method. As shown in Figure S2, the miRNA signature associated to mdx muscle fibres was confirmed by qRT-PCR analyses (Figure S2). Basing on this evidence, forthcoming quantification of miRNAs was performed only by Q-PCR analyses.

### Expression of Muscle-fibres Associated miRNAs during Disease Progression of the Mdx Mouse

**Figure 2:**
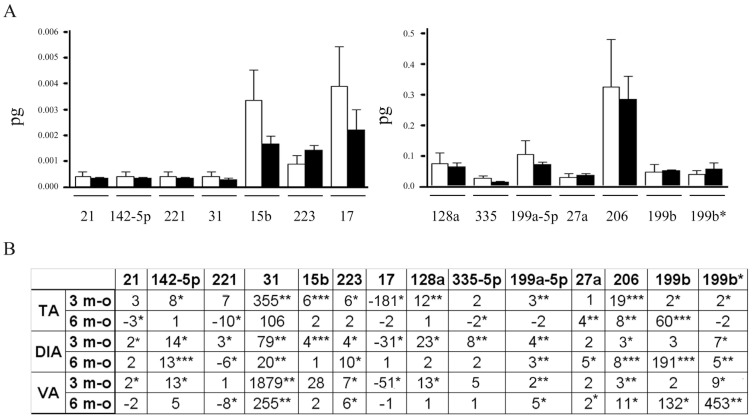
Dystrophin absence is not responsible for the over-expression of several muscle-enriched miRNAs in the mdx mouse. (A) MiRNAs that were observed over-expressed in the adult mdx mouse (3½ months-old) by the array analysis were quantified by Q-PCR in single fibres isolated from the hind limb of newborn mdx (n = 10) and c5bl (n = 10) mice. The absolute quantity (pg) of each miRNA was represented in the histogram as white columns for single fibres of c57bl mice and black bars for dystrophic muscle fibres. Single fibres of newborn mdx mice were characterized by control levels of muscle-enriched miRNAs, demonstrating no correlation with the genetic defect of DMD. (B) MiRNAs that were observed over-expressed in the adult mdx mouse (3½ months-old) by the array analysis were quantified by Q-PCR in single fibres isolated from the TA, DIA and VA of 6 months-old mdx mice and normalized on control samples. In the table were represented the fold change values of tested miRNAs in myofibres of 3½ and 6 months-old mdx mice in comparison to c57bl myofibers. Tested miRNAs showed a heterogeneous behaves depending on the muscle type and/or disease progression. (Two-wail parametric t-test; p value <0, 05 **p value <0, 01 ***p value <0,001).

In order to verify if the miRNA-dysregulation observed in single fibres of adult mdx mouse (3½ months-old) was associated to dystrophin absence, we isolated single muscle fibres from newborn mdx mice, which exhibit a better histological pattern than older 6 month-old mdx mice [35]. The mdx mouse is in fact characterized by progressive muscle deterioration due to increasing muscle loss and overcoming fibrosis [36]. Single fibres were dissected from the hind limb of newborn mdx (n = 10) and c57bl (n = 10) mice and from TA, DIA and VA of 6 moth-old mdx mice (n = 10). The expression levels of miRNAs were evaluated by absolute Q-PCR analysis. The statistical analysis did not evidence dysregulated miRNAs in newborn mdx muscle fibres, demonstrating any correlation with the genetic defect causing DMD and a strong association with accumulating degenerative/regenerative events (Figure 2B) Otherwise, analyses performed on older mdx mice showed a heterogeneous dysregulation of miRNAs. In particular, some miRNAs decreased to control levels in old mdx mice (miR-15b, miR-17, miR-31 and miR-128a) (Figure 2B), suggesting a major involvement in compensatory mechanisms activated by the muscle in the early step of disease. The expression levels of miR-21, miR-142-5p, miR-199a-5p, miR-199b*, miR-206, miR-223 and miR-335-5p were instead strictly related to the type of muscle considered, underling a relationship with muscle-type dependent impairment (Figure 2B). Finally miR-27a was found over-expressed in all muscle fibres analyzed (Figure 2B) and was recently demonstrated to down-regulate the expression of 2 fundamental myogenic transcription factors (paired box proteins PAX3 and PAX7) involved in the regulation of myogenesis and muscle development [37]. Interestingly the expression profile of miR-199b was shown parallel to disease progression, suggesting a strong correlation with the quality of resting dystrophic muscle or with accumulating muscle damage and fibrosis. Finally, miR-221 was characterized by an opposite trend of expression in adult (3½ m-o) and old (6 m-o) mdx mice respect control mice suggesting a double function of this miRNA during DMD pathogenesis (Figure 2B) which could be related to the muscle commitment and differentiation, as previously described [38].

### Chronic Damage Activates the miRNA Machinery in MDs

**Figure 3:**
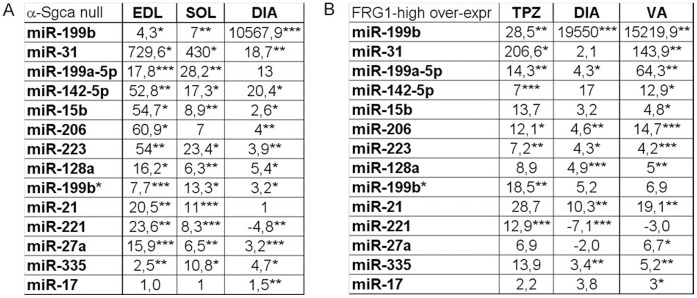
Different MDs are characterized by the same muscle-enriched miRNA-dysregulation. MiRNAs that were observed over-expressed in the adult 3½ months-old mdx mouse by the array analysis were quantified by absolute Q-PCR in single fibres isolated from EDL, SOL and DIA of α-Sgca null mice (A) and in single fibres isolated from TPZ, DIA and VA of FRG1 high over-expressing mice (B). MiRNAs average fold changes in muscle fibres of α-Sgca null mice (A) and of FRG1 over-expressing mice were shown in the tables in a linear scale. Tested miRNAs were similarly dysregulated in the animal model of different MDs. (Two-tail parametric t-test; *p value <0, 05; **p value <0, 01; ***p value <0,001).

Since dystrophic muscle fibres isolated from the mdx mouse exhibit a miRNA dysregulation that does not correlate with the genetic defect and is strictly related to muscle remodelling (resting fibres) and regeneration (newly-formed muscle fibres), we expected to observe a similar dysregulation in the animal models of other MDs. In this optic, we evaluated the previously tested miRNAs in an animal model of LMGD-2D (the α-Sgca null mouse) and with dystrophy similar to FSHD (the FRG1 high over-expressing mouse). Mice were chosen age- and sex-matched and the analysis included single muscle fibres isolated from different muscle types. We selected the less and the most affected muscles depending on the animal model: for the α-Sgca null mouse, we considered respectively soleus (SOL) and the extensor digitorum longus (EDL); for the FRG1 over-expressing mouse we analyzed respectively VA and TPZ. Furthermore, since DIA is mildly compromised in both animal models and was previously analyzed for the mdx mouse, we also included this muscle in the analysis of both animal models. MiRNA-expression levels were quantified by absolute Q-PCR analysis. The statistical analysis confirmed the miRNA-dysregulation observed in the mdx muscle fibres in single muscle fibres isolated from SOL, EDL and DIA of α -Sgca null mice. The only exceptions are represented by miR-206, miR-199a-5p and miR-17 whose dysregulation depended on the muscle considered (Figure 3A). In the case of the FRG1 over-expressing mice, only 4 of the tested miRNAs were found over-expressed in all muscle analyzed (miR-206, miR-223, miR-199a-5p and miR-199b), while the remaining 10 miRNAs showed a heterogeneous behaviour depending on the muscle considered. In particular, the dysregulation was limited to miR-199b*, miR-31, miR-142-5p and miR-221 in dystrophic TPZ; to miR-128a, miR-21, miR-221 and miR-35-5p in dystrophic DIA; and to miR-15b, miR-17, miR-27a, miR-142-5p, miR-128a, miR-335-5p, miR-21, miR-31 in dystrophic VA (Figure 3B). Basing on data mentioned above, the muscular miRNAs here evidenced are independent from the genetic defects of MDs and strongly correlate with chronic damage. The only exception is down-regulation of miR-17 that is specific of dystrophic single muscle fibres isolated from adult mdx mouse, suggesting its involvement in compensatory mechanisms triggered in the early-stage disease of this animal model of DMD. In fact, newborn mdx mice were characterized by normal expression levels of miR-17.

### Acute and Chronic Muscle Damage Recruits Different Set of miRNAs in Single Muscle Fibres with a Common Over-expression of miRNA 206

In order to further investigate muscle damage and to correlate miRNAs to muscle regeneration, we experimentally produced a controlled skeletal muscle injury by injecting CTX into TA of C57BL mice. By this approach, the induced damage mainly causes necrosis and consequent regeneration of fibres. Control mice (n = 10) were injected at day 0 and sacrified at 2, 5, 7, 10 days after injury. Before 2 days after CTX delivery, mice were not sacrified because there is evidence of extensive myonecrosis and edema of muscle fibres, which makes impossible the isolation of single fibres and the avoidance of non muscle cell contamination. Within 2 days a hypercellular response is observed, muscle regeneration is evident within 5 days from injury and by day 10 the architecture of the muscle is largely restored [39]. Since MDs are considered two-tired diseases where acute muscle damage is accompanied by low chronic background damage [14], we restricted the analysis to miRNAs previously associated to dystrophic muscle fibres. Single muscle fibres were isolated from injected and controlateral TA at each time point and miRNAs were quantified by absolute Q-PCR analyses. Data obtained evidenced a group of miRNAs whose expression does not change during muscle repair afterwards acute damage (miR-15b, miR-17, miR-128a, miR-221, miR-199a-5p miR-199b and miR-199b*) (Table 1), and a group of miRNAs that are triggered afterwards CTX delivery (miR-206, miR-31, miR-21, miR-335-5p, miR-27a, miR-142-5p and miR-223) (Table 1), suggesting major involvement of the latter in muscle regeneration. Interestingly the expression of miR-199b was not triggered by acute muscle damage, further supporting the strong correlation between its expression levels and the accumulation of chronic muscle damage, as previously demonstrated in the mdx mouse (Figure 2B). Moreover, miR-17 was not dysregulated afterwards acute muscle damage, confirming its specific role in the muscle pathophysiology of the mdx mouse. More importantly, miRNAs recently associated to muscle regeneration such as miR-31, miR-206 and miR-335-5p were confirmed over-expressed after acute damage [16].

**Table 1:** Acute muscle damage activates miRNA machinery in isolated myofibers.

	2d	5d	7d	10d
**miR-206**	−1,2	18,2	26,8**	4,5*
**miR-31**	1	19,5	14,2	9,6***
**miR-21**	2,7	5,5	12,7**	2,3*
**miR-335-3p**	1	3,1	8,9*	1,1
**miR-27a**	1,4	2,7	4,9**	1,7
**miR-142-p**	3,7*	6,5	8,6	1
**miR-223**	1	2,6**	1,4	−1,8
**miR-15b**	1,1	1,9	1,4	−1,7
**miR-17**	1,3	1,1	1,1	1,2
**miR-128a**	−2	−1,1	−1	−3
**miR-221**	−1,9	−2,8	−2	−2,8
**miR-199a-5p**	−2,7	1,3	2,2	−1,3
**miR-199b**	−1,3	1,1	1,4	1
miR199b*	1,1	−1,3	−1,2	−1,7

Dystrophic-fibres associated miRNAs were quantified by Q-PCR in single fibres isolated from TA of c57bl mice (n = 10) after CTX-injection. Mice were sacrified at the day 2, 5, 7, 10 after CTX-injury. In the table are represented the fold change values of tested miRNAs in CTX-injected muscle compare to controlateral not injected TA. MiR-206, miR-31, miR-21, miR-335-5p, miR-27a, miR-142-5p and miR-223 were significantly up-regulated afterwards muscle damage respect damaged muscle. Otherwise the other 7 miRNAs were not triggered upon CTX-injection.

(Two-tail parametric t-test; *p value <0, 05; **p value <0, 01; ***p value <0,001).

### New Muscular miRNAs and miRNA-206 are Over-expressed in DMD Patients

**Figure 4:**
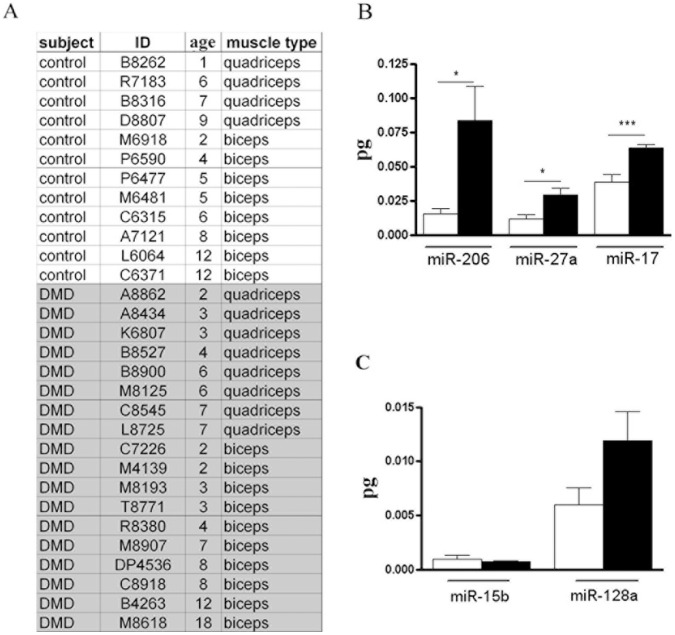
Identification of new murine dystrophic-fibres associated-miRNAs in muscle biopsies of DMD subjects. (A) The age and type of muscle of healthy subjects (12) and DMD patients (18) were listed. Moreover the type of muscle from which the biopsies were isolated were also reported. (B–C) MiR-15b, miR-128a, miR-206, miR-17 and miR-27a were quantified by absolute Q-PCR in muscle biopsies of control and dystrophic subjects listed in A. The absolute values (pg) of tested miRNAs were represented in the histogram for healthy (white bars) and dystrophic single fibres (black bars). (B) Quantitative analysis showed that only miR-128a, miR-206 and miR-17 were up-regulated in human dystrophic single fibres independently of the muscle type. (Two-tail parametric t-test; *p value <0, 05; **p value <0, 01; ***p value <0,001).

In agreement with data already published characterizing the miRNome of mdx and DMD muscle [15], [16], [17], the over-expression of several miRNAs (miR-21, miR-31, miR-199a-5p, miR-199b, miR-142-5p, miR-221, miR-223 and miR-335-5p) was confirmed in murine dystrophic single muscle fibres. Otherwise, single muscle fibres based-analyses highlighted new miRNAs (miR-17, miR-27a, miR-128a and miR-199b*) associated to dystrophic and/or damaged muscle. To verify reliability and clinical relevance of data obtained from the analysis of murine dystrophic single muscle fibres, we quantified the expression levels of miR-17, miR-27a and miR-128a in single muscle fibres dissected from human biopsies of DMD patients. Since miR-199b* is mouse-specific, the expression of this miRNA was not considered for the analyses. Moreover, two further miRNAs were quantified in human dystrophic fibres: miR-15b, whose over-expression was formerly observed in the mdx mouse by Cacchiarelli et al. [17] but not in DMD muscle [15]; and miR-206, whose expression profile was widely investigated in dystrophic and damaged muscle with controversial results [15], [16], [17], [34], [40]. Single muscle fibres were dissected from the muscle biopsies of 12 healthy subjects and 18 DMD patients with an age range between 1 and 18 y-o (Figure 4A), and expression levels of miR-15b, miR-17, miR-27a, miR-128a and miR-206 were quantified by absolute Q-PCR analyses. Data obtained confirmed over-expression of miR-17, miR-27a and miR-206 in single fibres of DMD muscle biopsies (Figure 4B and 4C). Otherwise the expression levels of miR-15b and miR-128a were similar in control and DMD human muscle fibres (Figure 4B and 4C), as already shown by Eisenberg et al. [15]. It is important to notice that miR-17 is down-regulated in muscle fibres of the mdx mouse but over-expressed in single fibres of DMD patients, further supporting the involvement of epigenetic during chronic muscle damage. Moreover the up-regulation of miR-206, which is a myomiR associated to muscle regeneration, was confirmed in human dystrophic fibres as already demonstrated by Greco et al [16].

### The Regeneration-associated myomiR-206 Down-regulates a Member of the High-mobility Group Proteins

**Figure 5:**
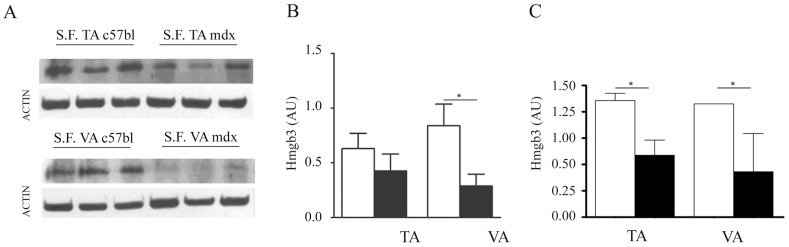
Hmgb3 expression-pattern in single fibres. (A) Immunoblotting experiments confirmed the presence of Hmgb3 protein in single muscle fibers isolated from the TA and VA of c57bl (n = 3) and mdx (n = 3) mice. (B) Densitometric analysis on WB bands evidenced a decreased expression of Hmgb3 in the TA and VA (p = 0.0133) of mdx mice. (C) Quantification of Hmgb3 mRNA by qRT-PCR in single fibres isolated from the TA and VA of mdx mice (n = 10) and of c57bl (n = 10) mice confirmed a down-regulation of Hmgb3 in dystrophic muscle. (Two-tail parametric t-test; *p value <0, 05; **p value <0, 01; ***p value <0,001).

**Figure 6:**
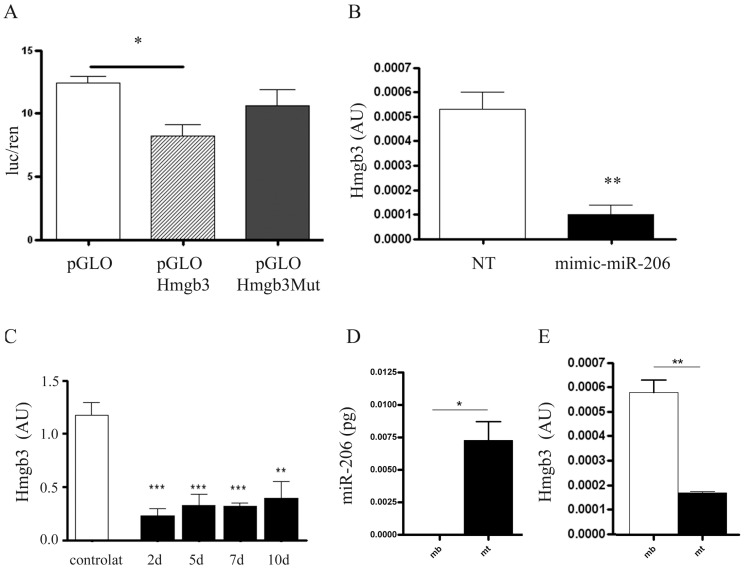
MyomiR-206 modulates Hmgb3 expression during myogenesis. (A) Hek cells were co-transfected with mimic-miR-206 at a concentration of 25 nM for 48 h and with pGLO-control or pGLOHmgb3 or pGLOHmgb3Mut. MiR-206 down-regulated the expression of luciferase of 32% (p = 0, 0151) in Hek cells co-transfected with pGLOHmgb3. Instead no down-regulation happened when the binding site of miR-206 was mutated. (B) 3T3 cells were transfected with mimic miR-206 at a concentration of 25 nM for 48 h, evidencing a strong down-regulation of endogenous Hmgb3 mRNAs. (C) Quantification of Hmgb3 mRNA by qRT-PCR in single fibres isolated from CTX-injured TA at day 2, 5, 7 and 10 from the injection evidenced a strong down-regulation of Hmgb3 during muscle regeneration. (D–E) Hmgb3 mRNA (D) and miR-206 (E) were respectively quantified by relative qRT-PCR and by absolute Q-PCR in proliferating C_1_C_12_ myoblasts cell line (MB) versus differentiated C_1_C_12_ myotubes (MT). The quantification analysis highlighted an opposite expression trend for Hmgb3 and miR-206, further validating inhibition of Hmgb3 by miR-206 (AU = arbitrary units). (Two-tail parametric t-test; *p value <0, 05; **p value <0,01; ***p value <0,001).

In order to localize myomiR-206 shown to be up-regulated in single muscle fibres of animal models of MDs and after acute damage, we performed i*n situ* hybridization experiments on tissue slices of TA isolated from the mdx and CTX-injected C57bl mice (3½ months-old). As shown in Figure S3, over-expression of myomiR-206 was limited to newly formed or regenerating fibres both in dystrophic and CTX-injected TA, confirming previous publications [33], [34]. These data are also in agreement with previous works describing several target genes of miR-206 such as the Connexin43, DNApolI, Utrophin and Pax7 during muscle regeneration [41], [42], [43], [44]. A target-gene prediction analysis was performed for myomiR-206 using 6 different computational algorithms (PITA, TargetScan, PicTar, ElMMo, miRDb and miRanda). Among the predicted target genes, we found the high mobility group box 3 (Hmgb3 or HMG4). In particular the potential binding of miR-206 to the 3′UTR of Hmgb3 involves two binding sites (first site goes from nt 575 to nt 585 of Hmgb3-3′ UTR; the second site goes from nt 714 to nt 726 of Hmgb3-3′UTR). Hmgb3 is a non-histone, chromatin-associated nuclear protein that interacts with transcription factors, enhancing the binding of these transcription factors to DNA and enhancing their transcriptional activity [30]. Its expression in adult mice was majorly observed in the bone marrow, where it regulates the balance of haematopoietic stem cells (HSCs) self-renewal [45], [46]. Furthermore Nemeth et al. highlighted an activated Wnt signalling in Hmgb3^−/−^ HSCs, suggesting an involvement in the regulation of Wnts members which are fundamental in muscle development, regeneration and myogenesis [47]. Since the expression of Hmgb3 was not previously investigated in skeletal muscle, we verified by Western Blot (WB) and qRT-PCR analyses its expression in single muscle fibres isolated from TA and VA of 3½ m-o c57bl mice. Surprisingly, Hmgb3 protein and mRNA were both easily detectable in single muscle fibres isolated from adult c57bl mice (Figure 5A and 5B). In order to verify if Hmgb3 can be modulated by myomiR-206, we performed functional tests in Hek cells using a standard luciferase assay. We cloned the 3′UTR of Hmgb3 downstream to the Firefly Luciferase reporter gene in the pmirGLO Dual-Luciferase Expression Vector (Promega, Madison, WI, USA) (PGLO), generating a plasmid (PGLO-Hmgb3) where the activity of the Firefly Luciferase is under the control of the 3′UTR of Hmgb3. The PGLO contains a constitutively expressed Renilla luciferase gene that is used to normalize the expression of the Firefly Luciferase, since it is an indicator of the transfection efficiency. We transiently co-transfected Hek cells with PGLO-Hmgb3 construct and the relative agonist of miR-206 (mimic-miR-206) (Thermo scientific, Waltham, MA, USA) at a concentration of 25 nM for 48 h. These experiments evidenced down-regulation (32%; p value = 0, 0151) of luciferase expression by co-transfection of Hek cells with PGLO-Hmgb3 and mimic-miR-206 (Figure 6A), confirming the target prediction. In order to support these results, we mutated the binding sites of myomiR-206 (n = 2) in the previously cloned 3′UTR of Hmgb3 and inserted the mutated 3′UTR of Hmgb3 in PGLO (Promega, Madison, WI, USA) (PGLO-Hmgb3-Mut). Hek cells were co-transfected with PGLO Hmgb3Mut and mimic-miR-206 (25 nM) for 48 h. In these conditions luciferase expression was not significantly down-regulated compared to PGLO-Hmgb3 transfected cells (Figure 6A). The same data were obtained when 3T3 cells, which endogenously express Hmgb3 (data not shown), were transfected with mimic-miR-206 (25 nM) for 48 h and the expression level of endogenous Hmgb3 was quantified by qRT-PCR analysis (Figure 6B). Since myomiR-206 is strongly induced in dystrophic single muscle fibres, we verified Hmgb3 down-regulation in dystrophic fibres by performing western blot analyses on muscle fibres isolated from adult mdx and control mice (Figure 5A). As shown in figure 5B, densitometric analysis on WB bands evidenced a decreased expression of Hmgb3 protein in TA and VA (p = 0.0133) of mdx mice. The same samples were tested for expression levels of Hmgb3 transcript, confirming down-regulation of Hmgb3 in dystrophic muscle fibres (Figure 5C). Since myomiR-206 is strongly induced during muscle regeneration and localizes in regenerating fibres (Figure S3) [17], [34], Hmgb3 mRNA was quantified in single fibres of CTX-injected muscle vs. controlateral un-injected TA. These experiments highlighted a strong down-regulation of Hmgb3 upon muscle injury (Figure 6C), concurrent with induction of myomiR-206 (Table 1), and the involvement of this target gene during muscle regeneration. To further verify the role of Hmgb3 in myogenesis, we quantified and compared Hmgb3 transcript and miR-206 expression in proliferating C_2_C_12_ myoblasts (MB) versus differentiated C_2_C_12_ myotubes (MT) obtained after 7 days of serum-privation (Figure 6D–E). These experiments evidenced opposite expression profiles of Hmgb3 and miR-206 during myogenesis. In particular, myotubes were characterized by low levels of Hmgb3 and high levels of miR-206 (Figure 6D and 6E). Taken together, these data demonstrate that up-regulation of miR-206 upon chronic and acute muscle damage down-regulates Hmgb3 and modifications of the chromatin-status can be speculated as down-stream event. Nevertheless, specific target genes of Hmgb3 need to be identified in order to more finely characterize the molecular pathway regulated by miR-206 through Hmgb3 upon muscle regeneration.

## Discussion

**Figure 7:**
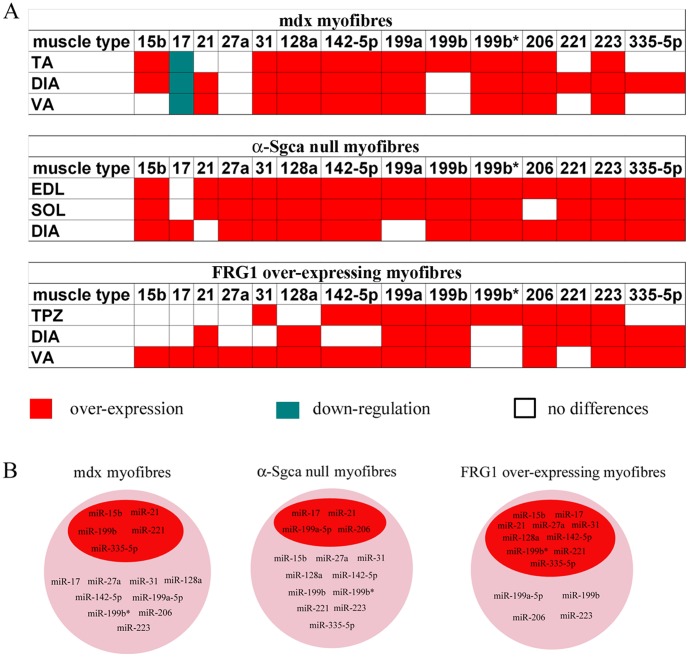
Expression profile of miRNAs associated to dystrophic single muscle fibres. (A) The expression profiles of miRNAs associated to dystrophic fibres of mdx, α-Sgca null and FRG1 over-expressing mice are reported as over-expressed (red block), down-regulated (green block) or similarly expressed (white block) to normal C57/BL fibres. (B) MiRNAs whose dysregulation in dystrophic fibres of mdx, α-Sgca null and FRG1 over-expressing mice depended on the muscle-type are listed in the red circle. Otherwise, miRNAs that were mis-modulated in dystrophic fibres of mdx, α-Sgca null and FRG1 over-expressing mice independently to the muscle-type are listed in the pink circle.

Following the discovery of miRNAs, their participation was investigated in almost all biological processes and, even more importantly, their central role in gene-expression regulation was implicated in many human diseases [3], [4], [5], [6], [7], [8] Regarding this, in the recent years many efforts were focused to finely characterize the role of miRNAs in myogenesis, so that now the miRNA biogenesis is considered necessary for proper muscle development and a restricted number of miRNAs, known as myomiRs (miR-1, miR-133, miR-181, miR-206, miR-208), is considered as integral part of muscle biology [9], [11]. Starting from these evidences, Eisenberg et al. demonstrated that muscle biopsies affected by 10 major primary muscular disorders are characterized by the dysregulation of several miRNAs, opening the possibility to use these small RNA molecules as therapeutic targets or potential biomarkers [15]. Nevertheless more investigation is required to finely tune myogenesis through the modulation of miRNAs. With the present study we wanted to identify miRNAs that are modulated by muscle fibres in response to damage in order to aloud muscle remodelling and regeneration. This purpose was reached by analysing the miRNome of muscle fibres isolated from dystrophic and CTX-injected muscle. Dystrophic muscle is characterized by progressive muscle wasting and weakness, representing an animal model of chronic muscle damage, while CTX-injected muscle is an animal model of acute damage. Importantly single-fibre based-analysis avoided contamination from non-muscle tissue, focusing the investigation on resting (remodelling) and newly-formed (regenerating) muscle fibres. In the present study three animal models of MDs were taken in consideration: the mdx mouse, animal model of DMD; the α-Sgca null mouse, animal model of LMGD-2D; and the FRG1 high over-expressing mouse, animal model with a dystrophy similar to FSHD. More in particular, we started by the miRNome of single fibres isolated from different muscles (TA, DIA and VA) of 3½ months-old mdx mouse. The dysregulated miRNAs associated to the mdx mouse were then verified during disease progression of this animal model and in single fibres of α-Sgca null mice (SOL, EDL and DIA) and FRG1 over-expressing mice (TPZ, DIA and VA) in order to evaluate their correlation with genetics of tested dystrophic mice. Dystrophic muscle fibres isolated from different animal model of MDs were commonly characterized by the over-expression of several miRNAs (miR-15b, miR-21, miR-27a, miR-31, miR-128a, miR-142-5p, miR-199a-5p, miR-199b, miR199b*, miR-206, miR-221, miR-223 and miR-335-5p) with an expression profile strictly dependent on muscle impairment and damage accumulation (Figure 7). In support to this, when the dysregulated miRNAs were analyzed during disease progression of the dystrophic mdx mouse (newborn, 3½ and 6 months-old), their expression levels were found similar to control levels in newborn mdx mice and heterogeneously modulated among the different muscles of older mdx mice, reflecting the heterogeneous distribution of muscle impairment. Taken together, these evidences also demonstrate no link between dystrophic miRNA-profile and the genetic causative defects of the different MDs, excluding the possibility to use any of the muscular miRNAs mentioned above as biomarker of a specific MD. Otherwise the over-expression of these miRNAs might function as a biomarker of chronic muscle damage. Surprisingly, apart from the regeneration-associated myomiR-206, no known myomiRs were found dysregulated in dystrophic single muscle fibres. Otherwise a group of miRNAs recently correlated to myogenesis (miR-27a, miR-31, miR-221) [33], [37], [38] were confirmed to be mis-modulated in dystrophic single muscle fibres. In comparison with data already published by Eisenberg et al. and Greco et al. [15], [16], single-fibres based-analysis allowed the discovery of new muscular miRNAs whose expression levels are up-regulated in dystrophic murine muscle (miR-15b, miR-27a, miR-128a and miR-199b*). For this reason we verified their expression in single muscle fibres isolated from biopsies of DMD patients. Analyses were also extended to myomiR-206 since its up-regulation in human dystrophic muscle was only confirmed by Greco et al. and not by Eisenberg et al. [15], [16]. MiRNAs were quantified in human muscle fibres of 12 control subjects and 18 DMD patients, confirming the over-expression of miR-17, miR-27a and miR-206 in diseased muscle.

In order to investigate the relationship between miRNAs associated to dystrophic muscle and muscle regeneration, we induced a controlled skeletal muscle injury by injecting CTX in TA of control mice. Only 7 of the 14 miRNAs associated to dystrophic fibres (miR-206, miR-31, miR-21, miR-335-5p, miR-27a, miR-142-5p and miR-223) were triggered by CTX injury. We hypothesized that acute CTX damage triggers the expression of these miRNAs, while chronic-damage associated-miRNAs may be activated by other compensatory mechanisms participating to deterioration of resting dystrophic fibres. MyomiR-206 is one of the most investigated myomiRs in skeletal muscle tissue [40]. Like miR-133b, its expression is regulated by MEF2 and MyoD [41], [42]. It was found to promote muscle differentiation by, in part, inhibiting a component of DNA polymerase [41] as well as follistatin-1 (Fstl1) and utrophin (Utrn), genes that are suppressed in fibroblasts converted to skeletal muscle cells [42]. It was also shown to regulate skeletal muscle satellite cell proliferation and differentiation by repressing paired box gene 7 (Pax7), which is fundamental for regulation of myogenesis [44]. As previously described [17], [34] we demonstrate dysregulation and in situ localization of miR-206 in regenerating single fibres. Nevertheless the big limitation in understanding its biological function and application in the field of therapeutics, lies on only few validated target genes that are downstream players in important pathways. For these reasons we performed a prediction-analysis of target genes and looked for regulators of epigenetic and Hmgb3 was one of the listed gene. Hmgb3 is a member of the HMG superfamily which is the most abundant non-histone proteins in the eukaryotic nucleus [30]. In particular it is classified with Hmg-3 and -2 into Hmg-box subfamily, defined by the presence of DNA-binding Hmg-box domains [30]. Up to now its expression was majorly localized to the bone-marrow where it regulates the balance of HSC, through modulation of Wnt-signalling [45]. In particular Hmgb3^−/−^ HSCs were characterized by increased levels of Wnt signalling; members of the Wnt family are involved in various stages of skeletal muscle development and regeneration [47]. For this reason we investigated the expression of Hmgb3 in single fibres isolated from TA and VA of control mice and confirmed its expression in the skeletal muscle. Furthermore, experiments performed on muscle fibres isolated from dystrophic TA, VA and CTX-injected muscle, confirmed that Hmgb3 is down-regulated consequently to acute and chronic muscle damage mirrored to myomiR-206. Basing on these experimental evidences, we performed in-vitro luciferase assays confirming an inhibitory effect of myomiR-206 on Hmgb3. In order to further demonstrate the involvement of Hmgb3 in myogenesis, its expression levels were evaluated during differentiation of C_2_C_12_ into myotubes and a strong down-regulation of Hmgb3 was observed mirrored to myomiR-206. These results together with data already published demonstrating modulation of Wnt family members upon C_2_C_12_ cells differentiation [47], support a possible correlation of Hmgb3 expression levels with Wnt signalling in skeletal muscle, as already demonstrated for HSCs [45]. Furthermore, since Hmgb3 is non-histone, chromatin-associated nuclear protein that interacts with transcription factors enhancing their transcriptional activity, myomiR-206 might participate to muscle regeneration by sculpting the chromatin and regulating the transcription of several genes, like Wnts. In conclusion the present study provides a detailed image of muscular miRNAs that are expressed in single muscle fibres and are modulated in a condition of acute and chronic muscle damage. Importantly the analysis of single muscle fibres guaranteed no contaminations from surrounding tissues, enabling the identification of a subset of miRNAs that are actually linked to muscle remodelling and could be a target for recovery. Furthermore the analyses of single fibres enabled the discovery of new muscular miRNAs and did not evidence the dysregulation of many others, underlining the importance to accurately select the starting material for the analysis of miRNAs in biological processes. In this study we have also shown the expression of non-muscle enriched miRNAs, such as the granulocytes-associated miR-223 and haematopoietic miR-142-5p, in single muscle fibres. In agreement with our finding, previously published data demonstrated expression of miR-223 and miR-142-5p not limited to granulocytes or haematopoietic lineage. In particular, miR-223 was localized in cardiomyocytes, with prominent peri-nuclear staining, and in non-myocytes by in situ hybridization on cryosection of heart [48]; and miR-142-5p was detected in embryonic stem cells (ESC) where it is involved in cardiac differentiation [49]. Moreover expression of miR-223 and miR-142-5p in single muscle fibres might reflect the common mesodermic origin of muscle and hematopoietic tissues. In support to this: miR-335 and miR-21 were found in human mesenchymal stromal cells [50] and in mesenchymal stem cells (MSCs) together with miR-21, miR-27a, miR-128a, miR-199b [51] miR-15b, miR-17, miR-21, miR-27a, miR-31, miR-199a, miR-199b, miR-221 and miR-335-5p were found in MSCs and in MSC secreted microparticles [49], [52], [53]. In conclusion we suggest for the first time that Hmg-box family transcription factors, previously linked to biological hematopoietic circuits, is also part of the pathways controlling muscle regeneration. Identification of genes controlled by myomiR-206 through Hmgb3 can be instrumental to explain the molecular mechanisms involved in myomiR-206 mediated muscle regeneration in muscular dystrophy and muscle damage.

## Materials and Methods

### Ethics Statement

Mice were obtained from Charles River Laboratories (Calco, Italy), from Dr Cossu and Gabellini Labs (San Raffaele Hospital, Milan, Italy). All procedures involving living animals conformed to Italian Country law (D.L.vo 116/92 and subsequent additions). Human samples were collected after signed informed consent from all participants according to the guidelines of the Committee on the Use of Human Subjects in Research of the Fondazione IRCCS Ca’ Granda Ospedale Maggiore Policlinico di Milano (Milan, Italy) and after approval of the ethics committee of the University of Milan, Italy (CR937-G) which also authorized the use of animals for this study.

All cell lines were commercially obtained from ATCC: C2C12: ATCC number CRL-1772, NIH-3T3: ATCC number CRL-1658, and HEK293: ATCC number CRL-1573.

### Animal Models

Newborn-, 3½ m-o and 6 m-o dystrophic mdx mice and C57BL mice obtained from Charles River Laboratories (Calco, Italy), 4 m-o α-Sgca null mice given from Dr Cossu and 4 m-o old FRG1 over-expressing mice offered by Dr Gabellini were used throughout this study.

### Human Samples

Muscle biopsies from DMD (n = 18) and control subjects (n = 12) were collected. All dystrophic patients (n = 18) had a diagnosis of DMD based on the absence of dystrophin immunoreactivity on biceps and quadriceps muscle sections and based on PCR genotyping. None of the participants at the time of biopsy was treated with corticosteroid drugs. All control subjects included in this study presented at the time of the biopsy a suspect metabolic disorder not confirmed by biochemical and histopathological studies. Control biopsies did not show signs of muscle pathology on histological and histochemical examination. All bioptic specimens used in this study were taken, for diagnostic purposes, under institutionally approved protocols. Since all our patients were minors, their parents were asked to sign an informed consent disclosing future use of the bioptic material for research.

### Single Fibres Extraction

This study was approved by ethics committee of the University of Milan, Italy (CR937-G). Mice were killed by cervical dislocation and vastus (VA), diaphragm (DIA), tibialis (TA), extensor digitorum longus (EDL), soleus (SOL) and trapezius (TPZ) were carefully dissected away from the surrounding musculature. Single fibres were isolated by dissected muscles as described in Bischoff et al. [54]. From freshly isolated fibres, total RNA extraction was performed using TrizolReagent (Invitrogen, Life Technologies, Carlsbad, California, USA), as described the manufacturer’s protocol.

### MiRNA Array Analysis

Single muscles fibres were isolated from TA, DIA and VA of mdx (n = 3) and c57bl normal (n = 3) mice. Isolated muscle fibres were frozen in liquid nitrogen-cooled isopentane and shipped to Miltenyi Biotec (Bergisch Gladbach, Germany). RNA extraction of single myofibres, microarray data processing and analysis was performed by Miltenyi Biotech. (Bergisch Gladbach, Germany). Total RNA was isolated using TrizolReagent (Invitrogen, Life Technologies, Carlsbad, California, USA) as described the manufacturer’s protocol. The quality of total RNA samples was checked by applying the RNA Integrity Number (RIN) which is calculated by a proprietary algorithm of the Agilent 2100 Bioanalyzer expert software. As hybridization control the Universal Reference provided by Miltenyi Biotec GmbH was used. Fluorescence signals of hybridized PIQORTM Microarrays were detected using a laser scanner from Agilent (Agilent Technologies). Low-quality spots were flagged and excluded from data analysis. Unflagged spots were analysed with the PIQORTM Analyzer software. For statistical analysis the Two-class SAM (Significance Analysis of Microarrays) method was used. All data are MIAME compliant and raw data has been deposited in GEO database (http://www.ncbi.nlm.nih.gov/geo/query; accession number GSE21219).

### Quantification of miRNAs by Absolute (Q-PCR) and Relative (qRT-PCR) Real-Time PCR Analysis

RNA extraction was performed from single fibres isolated from TA, VA and DIA of mdx mice (3½ and 6 m-o) (n = 10); from EDL, SOL and DIA of α-Sgca null mice (n = 10); from VA, DIA, and TPZ of FRG1 over-expressing mice (n = 10); from single muscle fibres isolated from muscle biopsies of 12 control subjects and 12 DMD patients; from proliferating and differentiated C_2_C_12_ cell lines. Total RNA was extracted with TrizolReagent as described by the manufacturer’s protocol (Invitrogen Life Technologies, Carlsbad, California, USA). TaqMan MicroRNA Assays (Applied Biosystems, Austin, USA) was used to detect and accurately quantify mature miRNAs using PTC200 Chromo4 MJ Research instrument (Biorad Laboratories, Hercules, CA, USA). Aliquots of total RNA (10ng) were used to analyze miR-1, miR-133a, miR-181b, miR-15b, miR-17, miR-128a, miR-142-5p, miR-335-5p, miR-31, miR-206, miR-21, miR221, miR-223, miR-199a-3p, miR-199a-5p, miR-199b and miR27a by absolute Real-Time PCR (Q-PCR) using the TaqMan MicroRNA Assays (Applied Biosystems, Austin, USA). In order to obtain an absolute quantification, we synthesized RNA oligonucleotides (MWG-Biotech, Ebersberg, Germany) with the same sequence of muscle miRNAs that were listed above. 10 ng of the synthetic miRNAs were retrotranscribed and 10 serial dilutions (1∶5) of cDNA corresponding to 10 ng, 2 ng, 400 pg, 80 pg, 16 pg, 3.2 pg, 0.64 pg, 0.128 pg, 0.0256 pg and 0.0051 pg prepared from oligonucleotides were analyzed by Q-PCR. The calibration curve was used to obtain the quantity of miRNAs contained in each muscle sample. Samples were also tested for the expression of small RNA U6 (U6sn RNA) using the TaqMan MicroRNA Assays (Applied Biosystems, Austin, USA) in order to evaluate the quality and quantity of starting RNA: all tested samples differed maximum of two C_T_ when analyzed for RNAU6, demonstrating comparable quantity of starting RNAs. Furthermore, each time we performed an absolute Q-PCR analysis to evaluate the expression profiles of miRNAs, we also evaluated in the same plate the expression levels of RNAU6. Each experiment was performed three times and for each sample, the median value between the three experiments was reported. Analysis of statistical significance was determined by two-tail paired-t-test (Two-wail parametric t-test; p value <0, 05 **p value <0, 01 ***p value <0,001).

### Relative Quantification of Hmgb3 by Real-Time PCR (qRT-PCR)

For quantification of Hmgb3 and GAPDH, total RNA (1 µg) obtained from whole muscle, single fibres, proliferating and differentiated C2C12 cell line were treated with DNAse-RNAse free for 1 h at 37°C (Promega, Madison, WI, USA) in order to avoid amplification of genomic DNA. Dnase-treated RNAs were restrotranscribed by using Super Script First Strand Synthesis System III for RT-PCR (Invitrogen, Life Technologies) with oligo(dT)_16_ primers. cDNA (1.5 µl) was subjected to qRT-PCR using PTC200 Chromo4 MJ Research instrument (BioRad Laboratories, Hercules, CA, USA). qRT-PCR was performed in 20 µl volumes using FluoCycle SYBR Master Mix (Euroclone, Milan, Italy). All PCR assays were performed in triplicate and the data were pooled. Before using the ΔC_T_ method for relative quantification [55], we performed a validation experiment to demonstrate the efficiency of the primers of the target genes. The reaction conditions were as follows: 95°C for 10 min; followed by 50 cycles at 95°C for 15 s (denaturation) and 60°C for 1 min (annealing and elongation). After each experiment Melting Curve from 55.0°C to 95.0°C, ready every 0.5°C was performed. Threshold cycle numbers (C_T_) were determined using MJ OpticonMonitor Analysis Software and transformed using the ΔCT (2^−ΔC^_T_) comparative method [54]. The data were showed as normalized expression (2^−ΔC^_T_). Briefly, the ΔC_T_ value is determined by subtracting the average GAPDH C_T_ value from the average of the C_T_ value of the target gene for the same sample. Gene-specific expression values were normalized to expression values of GAPDH gene (endogenous control) within each sample. The sequences of the primer were.Mouse GAPDH F: 5′ GTGGCAAAGTGGAGATTGTTGCC 3′Mouse GAPDH R: 5′ GATGATGACCCGTTTGGCTCC 3′.Mouse HMGB3 F: 5′ CCGGCATGAGAAGTGTTTGGA 3′.Mouse HMGB3 R: 5′ TGCGTTTAGGTCCAGAGGGTT 3′.

### Acute Muscle Damage Experiments

For acute muscle damage assay, injury was performed in 3½ month-olds c57bl mice by injecting 100 µl of 10 µM CTX in TA, as previously described [25]. Mice were sacrificed at 2, 5, 7 and 10 days post-injection (10 mice per each time point). Serial sections of 10 µm in thickness were used for in-situ hybridization (ISH) and were stained with Hematossin & Eosin (H&E).

### In situ Hybridization

In situ hybridization was performed using a locked nucleic acid (LNA) detection probe for mmu-miR-206 (Exiqon, Vedbaek, Denmark), which was labelled with digoxigenin [31] using a DIG oligonucleotide tailing kit (Roche, Milano, Italy). Tissue slices of TA of 3½ month-olds mdx mice and CTX-injected tibialis of c57bl mice were fixed with 4% paraformaldehyde (PFA) in PBS, were immersed in 100% methanol, rehydrated, treated with proteinase K, and then re-fixed with PFA. After stringent wash at 55°C in 50% formamide, 2×SSC and then PBS, they were incubated with blocking solution, followed by alkaline phosphatase-conjugated anti-DIG antibody (Roche, Milano, Italy). Hybridized probes were detected and visualized by colour reaction with alkaline phosphatase substrate kit red (Vector Labs, Burlingame, CA, USA).

### Prediction of Potential Target Genes of the Differentially Regulated miRNAs

Using different computational approaches it is possible to predict potential binding sites for miRNAs in the genome, evidencing potentially regulated mRNAs. The predicted targets usually are identified based on the presence of a 6-8bp target site (or “nucleus”) for the respective miRNA mainly located in the 3′UTR of the mRNA. Although the various target prediction approaches consider additional features like evolutionary conservation of the target sites, structural target site accessibility, and minimum free energy of the local miRNA/mRNA interaction, the number of potential targets per miRNA is still high. The miRNAs with significant expression differences between dystrophic and wild type muscle samples were subjected to various target prediction algorithms which are publicly available (PITA, TargetScan, PicTar, ElMMo, miRDb and miRanda). Each of the algorithms returns several hundreds of potential targets for each miRNA which would sum up to several thousands potential targets of all differentially regulated miRNAs identified in this project.

### Evaluation of Hmgb3 Protein Expression by Western Blot Analysis

Single fibres and whole muscles were lysed directly in 1× sample buffer (1% SDS) containing 2 mg/ml aprotinin, 10 mg/ml leupeptin, 10 mM sodium fluoride (NaF), 1 mM sodium vanadate (Na_3_VO_4_) and 1 mM phenylmethylsulfonylfluoride (PMSF). Lysates were boiled 5 min and centrifuged at 10000×g for 5 min to remove insoluble material. Total protein concentration was determined according to Lowrys method and lysates were stored at −20°C. Samples were analyzed on 7.5% polyacrylamide gel, transferred to supported nitrocellulose membranes (BioRad Laboratories, Hercules, CA, USA), and the filters were saturated in blocking solution (10 mM Tris, pH 7.4, 154 mM NaCl, 1%BSA, 10% horse serum, 0.075%Tween-20) overnight at 4°C. Primary antibodies anti-Hmgb3 (RD systems Inc., Newcastle, USA) and anti-β-actin (Sigma, Milan, Italy) were incubated for 90 min at room temperature and then followed by washing, detection with horseradish peroxidase (HRP) conjugated secondary antibodies (DakoCytomation, Carpinteria, CA, USA), and developed by enhanced chemiluminescence (ECL) (Amersham Biosciences, Piscataway, NJ, USA). Prestained molecular weight markers (Bio-Rad Laboratories, Hercules, CA, USA) were run on each gel. Bands were visualized by autoradiography using Amersham Hyperfilm (Amersham Biosciences, Piscataway, NJ, USA).

### Luciferase Assay

The murine Hmgb3-3′UTR region was obtained by 3T3 cells. Total RNA was extracted using TrizolReagent as previously described (Invitrogen, Life Technologies, Carlsbad, California, USA) and first-strand cDNA was prepared starting from 2ug total RNA with 50 µM oligo-dT primers using Super Script III First Strand Synthesis System for RT-PCR (Invitrogen, Life Technologies, Carlsbad, California, USA). PCR reactions was carried out with a final mix constituted by 1× HF Phusion buffer, 0.2 mM dNTPs mix, 1 unit of the Phusion High-fidelity DNA Polymerase, 10 pMol of forward primer (5′CTCGAGGACACATCTCTTATTTGAGTC3′), and 10 pMol of reverse primer (5′GTCGACACATTTCAGCATTCTAAACATAC3′) containing a XhoI and SalI restriction site respectively. Thirty-five cycles of amplification were used. The PCR product was cloned into the pmiRGLO Dual-Luciferase Expression Vector (Promega, Madison, USA). The sequences were always verified by sequencing. The predicted miR seed sites were mutated using the QuickChange Multisite mutagenesis kit (Stratagene, Santa Clara, California, USA) with the following mutagenic primers:miR-206.1 F: 5′ GGCATAGCATTCTGCGGCCGGCATTAGTTGTGGAAAGG 3′miR-206.2 F: 5′ CCTAAACGCAATTTGCAGACGCATGTTATTTTTTGTATG 3′.

Mutations were confirmed by sequence analysis. The wild-type and mutated 3′′UTRs (respectively referred as pGLOHmgb3 and pGLOHmgb3-Mut) were subcloned into XhoI- SalI site of the pGLO (Promega, Madison, USA).

### Hek Transfection with mimic-miR-206 and Luciferase Assay

Hek cells were maintained in DMEM supplemented with 10% FBS and penicillin-streptomycin. Hek cells were seeded at 1.3×10^5^ cells per well of a 24-well plate and grown for 20 h. pGLOHmgb3 reporter plasmid or its mutants were co-transfected with mimic-miR-206 (Thermo scientific, Waltham, Massachusetts) at a final concentration of 25 nM using Lipofectamine 2000 (Invitrogen, Life Technologies, Carlsbad, California, USA). The transfection mixtures for each sample contained 2 µl of Lipofectamine (Invitrogen, Life Technologies, Carlsbad, California, USA), 25 nM of mimic (Thermo scientific, Waltham, Massachusetts) and 1.5 µg of pGLO or pGLOHmgb3 or pGLOHmgb3-Mut in 100 µl total volume of DMEM (Gibco, Life Technologies, Carlsbad, California, USA) without antibiotics and FBS (Gibco, Life Technologies, Carlsbad, California, USA). These reporter plasmids contained Renilla Luciferase gene that was considered for normalizing the transfection. Cells were collected 48 h after transfection, and luciferase activity was measured using a dual-luciferase reporter assay system with a GloMax luminometer (Promega, Madison, USA).

### 3T3 Transfections with mimic-miR-206

3T3 cells were seeded 1×10^5^cells per well of a 24-well plate and grown for 20 h in DMEM supplemented with 10% FBS and penicillin-streptomycin. The cells were transfected with mimic-miR-206 (Thermo scientific, Waltham, Massachusetts) at a final concentration of 25 nM using Lipofectamine2000 (Invitrogen, Life Technologies, Carlsbad, California, USA). 48 h after transfection, cells were collected and total RNA was extracted using TrizolReagent as previously described (Invitrogen, Life Technologies, Carlsbad, California, USA). Total RNA obtained from whole muscle and single fibres were treated with DNAse-RNAse free (Promega, Madison, WI, USA) in order to avoid amplification of genomic DNA. First-strand cDNA was prepared by using Super Script III First Strand Synthesis System for RT-PCR (Invitrogen, Life Technologies, Carlsbad, California, USA), starting from 1 µg total RNA with 50 ng random hexamer primers. Hmgb3 expression levels were quantified by qRT-PCR analysis in transfected and non-transfected 3T3 cells.

## Supporting Information

Figure S1 **Quantification of miRNAs by absolute Q-PCR analysis.** The miRNAs signature was confirmed by absolute Q-PCR analysis. The analysis was also extended to miR-1, miR-133 and miR-181. Expression profile of evaluated miRNAs in the TA (A), DIA (B) and VA (C) of 3½ months-old c57bl (white columns) and mdx mice (black columns) are shown. (Two-tail parametric t-test; *p value <0, 05; **p value <0, 01; ***p value <0,001).
(JPG)

Figure S2 **Quantification of miRNAs by relative qRT-PCR analysis.** The miRNAs signature was confirmed by relative qRT-PCR analysis. The analysis was also extended to miR-1, miR-133 and miR-181. Expression profile of evaluated miRNAs in the TA (A), DIA (B) and VA (C) of 3½ months-old c57bl (white columns) and mdx mice (black columns) are shown. (Two-tail parametric t-test; *p value <0, 05; **p value <0, 01; ***p value <0,001).
(TIF)

Figure S3 **Expression and localization of miR-206 in damaged and dystrophic muscle.** H&E staining (A) and miR-206 in situ hybridization (B), performed on serial sections of TA dissected from 3½ months-old mdx mice, are shown. H&E staining (C, E, G, I) and miR-206 ISH on serial sections (D, F, H and L) of CTX-injected control TA after day 2 (C, D), 5 (E, F), 7 (G, H) and 10 (I, L) after injury are reported. In situ hybridization analysis showed intense signals of miR-206 in newly formed muscle fibres with centralized nuclei, or regenerating fibres, in both models of muscle damage.
(TIF)
