# Supplementary figures and images for: Hmgb3 Is Regulated by MicroRNA-206 during Muscle Regeneration
Source: PLoS One. 2012 Aug 17;7(8):e43464. doi: 10.1371/journal.pone.0043464 (PMC3422271; doi:10.1371/journal.pone.0043464)

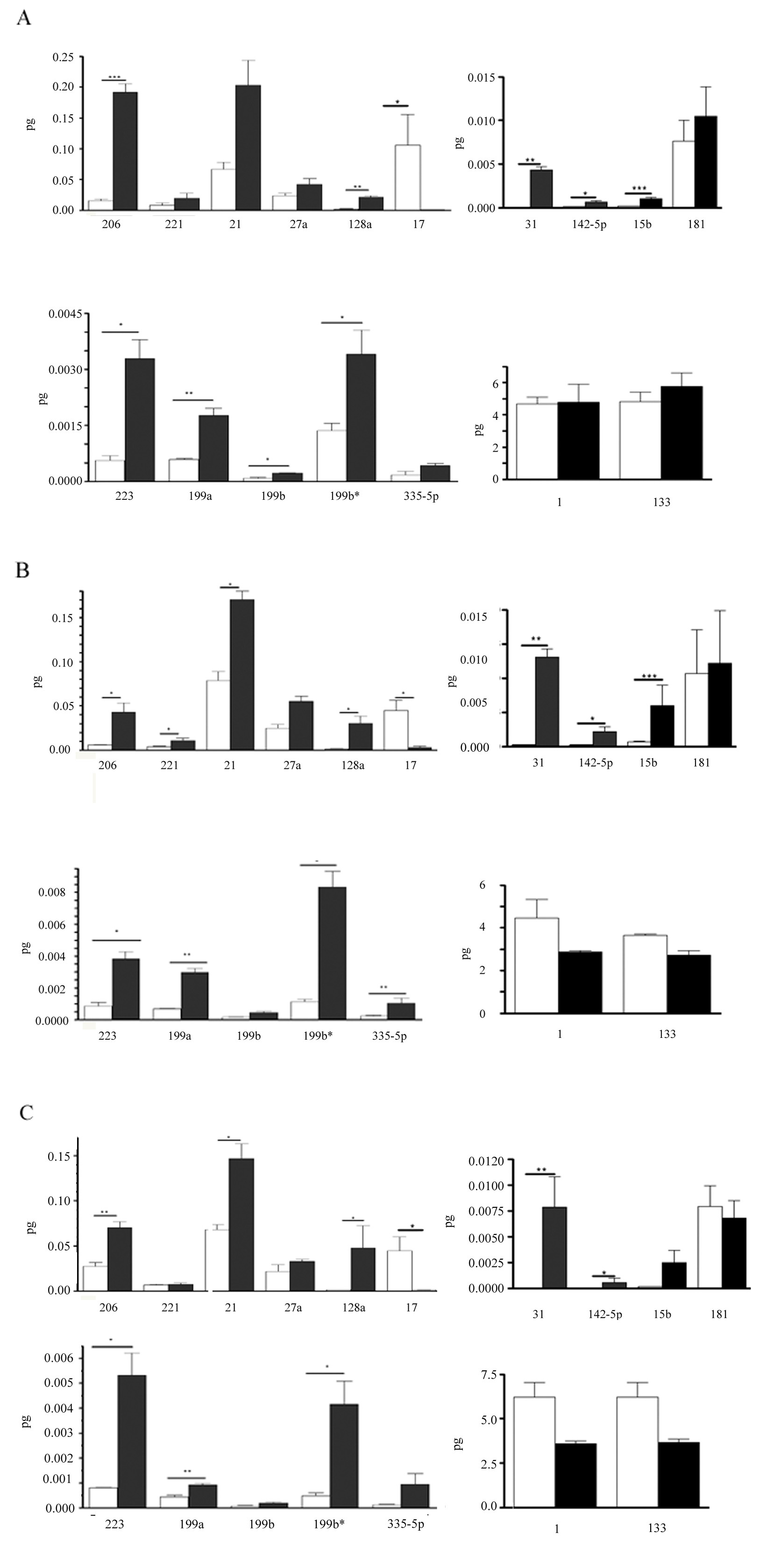

Supplement: Figure S1 — Quantification of miRNAs by absolute Q-PCR analysis. The miRNAs signature was confirmed by absolute Q-PCR analysis. The analysis was also extended to miR-1, miR-133 and miR-181. Expression profile of evaluated miRNAs in the TA (A), DIA (B) and VA (C) of 3½ months-old c57bl (white columns) and mdx mice (black columns) are shown. (Two-tail parametric t-test; *p value <0, 05; **p value <0, 01; ***p value <0,001). (JPG) [file pone.0043464.s001.jpg]

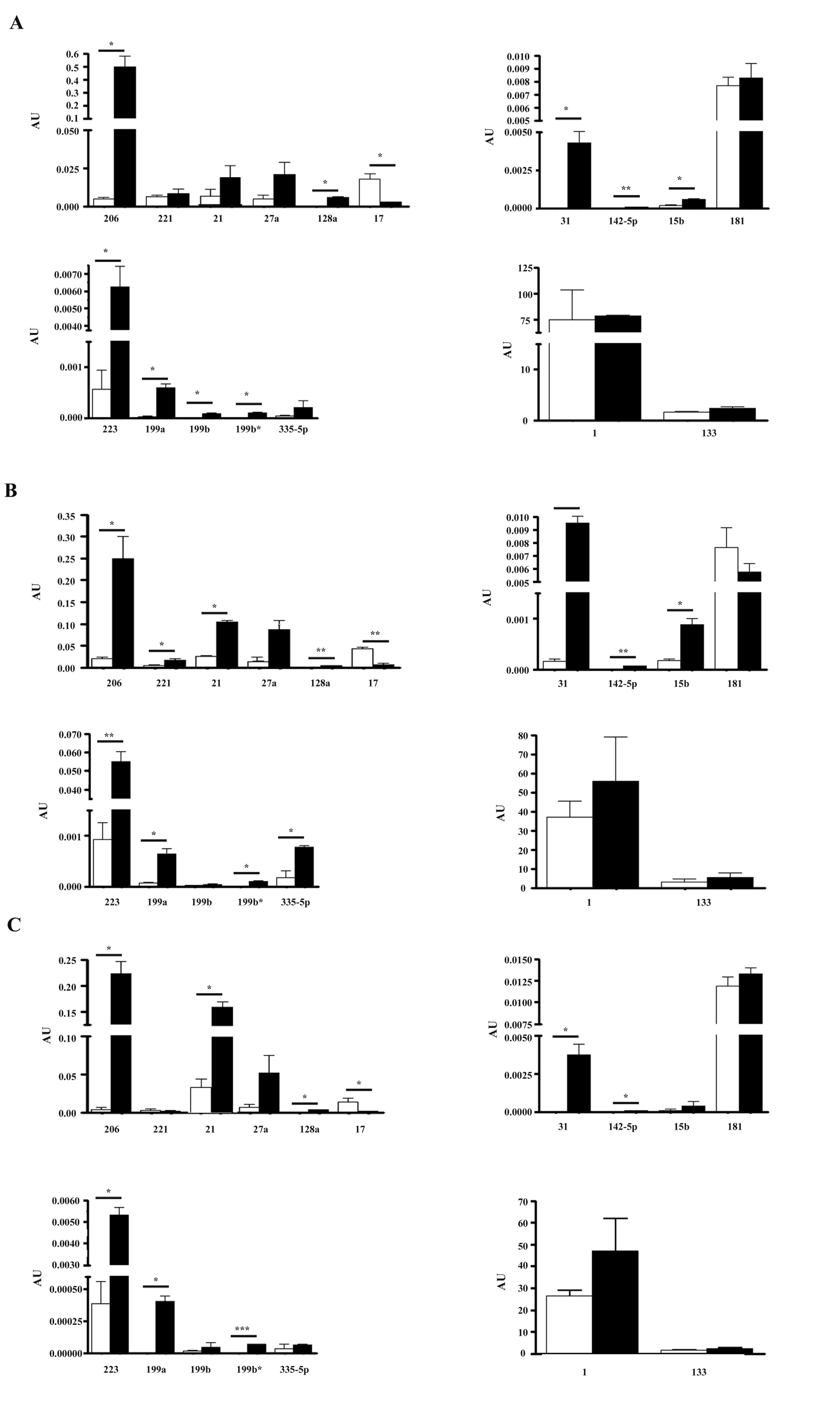

Supplement: Figure S2 — Quantification of miRNAs by relative qRT-PCR analysis. The miRNAs signature was confirmed by relative qRT-PCR analysis. The analysis was also extended to miR-1, miR-133 and miR-181. Expression profile of evaluated miRNAs in the TA (A), DIA (B) and VA (C) of 3½ months-old c57bl (white columns) and mdx mice (black columns) are shown. (Two-tail parametric t-test; *p value <0, 05; **p value <0, 01; ***p value <0,001). (TIF) [file pone.0043464.s002.tif]

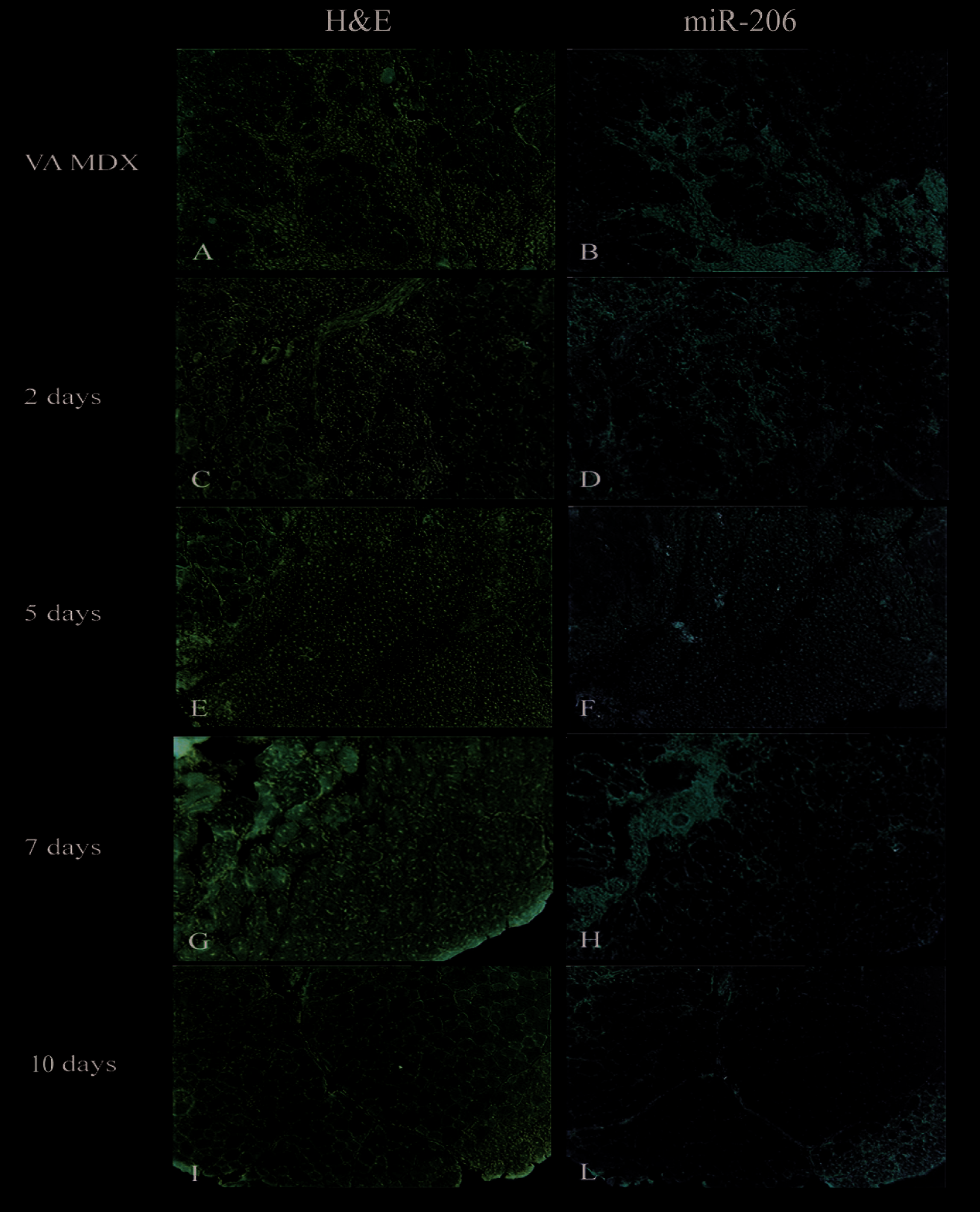

Supplement: Figure S3 — Expression and localization of miR-206 in damaged and dystrophic muscle. H&E staining (A) and miR-206 in situ hybridization (B), performed on serial sections of TA dissected from 3½ months-old mdx mice, are shown. H&E staining (C, E, G, I) and miR-206 ISH on serial sections (D, F, H and L) of CTX-injected control TA after day 2 (C, D), 5 (E, F), 7 (G, H) and 10 (I, L) after injury are reported. In situ hybridization analysis showed intense signals of miR-206 in newly formed muscle fibres with centralized nuclei, or regenerating fibres, in both models of muscle damage. (TIF) [file pone.0043464.s003.tif]
